# NicE-seq: high resolution open chromatin profiling

**DOI:** 10.1186/s13059-017-1247-6

**Published:** 2017-06-28

**Authors:** V. K. Chaithanya Ponnaluri, Guoqiang Zhang, Pierre-Olivier Estève, George Spracklin, Stephanie Sian, Shuang-yong Xu, Touati Benoukraf, Sriharsa Pradhan

**Affiliations:** 1 New England Biolabs Inc., 240 County Road, Ipswich, MA 01938 USA; 2 Cancer Science Institute of Singapore, National University of Singapore, Singapore, 117599 Singapore

**Keywords:** Open chromatin, NicE-seq, Transcription factor occupancy, DNA methylation

## Abstract

Open chromatin profiling integrates information across diverse regulatory elements to reveal the transcriptionally active genome. Tn5 transposase and DNase I sequencing-based methods prefer native or high cell numbers. Here, we describe NicE-seq (nicking enzyme assisted sequencing) for high-resolution open chromatin profiling on both native and formaldehyde-fixed cells. NicE-seq captures and reveals open chromatin sites (OCSs) and transcription factor occupancy at single nucleotide resolution, coincident with DNase hypersensitive and ATAC-seq sites at a low sequencing burden. OCSs correlate with RNA polymerase II occupancy and active chromatin marks, while displaying a contrasting pattern to CpG methylation. Decitabine-mediated hypomethylation of HCT116 displays higher numbers of OCSs.

**Electronic supplementary material:**

The online version of this article (doi:10.1186/s13059-017-1247-6) contains supplementary material, which is available to authorized users.

## Background

The mammalian genome is largely packaged into chromatin consisting primarily of DNA, proteins, and RNA. This macromolecular structure is further condensed into larger folded structures, such as chromosomes, during cell division. The cell cycle phase and the transcriptional status of the cell influences the state of the chromatin. It often undergoes remodeling events for switching between closed and open conformations to provide accessibility to DNA-binding proteins, including transcription factors [1–3]. In addition to core histones, chromatin contains a wide variety of non-histone chromosomal proteins involved in various activities, including DNA replication and gene expression [4, 5].

A series of genome-wide methods and approaches for mapping chromatin accessibility (open chromatin), nucleosome positioning, transcription factor occupancy, and other chromosomal protein binding have been established to decipher the epigenetic information encoded in the chromatin [6–14]. Early studies identified nucleosome-depleted regions as being hypersensitive to DNase I and point to the association of these protein-depleted regions with gene activation in eukaryotic organisms [15–18]. Although DNase I-based sequencing (DNase-seq) is a powerful method on its own, it requires specific reagents and vast amounts of cells. Mapping of open chromatin by DNase-seq requires between one and ten million cells and often involves titration of enzyme and multiple steps before the library is made for sequencing. DNase-seq employs the preferential digestion of nucleosome- and transcription factor-depleted sites using DNase I and it identifies DNase I hypersensitivity sites (DHSs) by sequencing [16, 17]. In another method, transcription factor binding sites are interrogated using chromatin-immunoprecipitation (ChIP) sequencing technology [19]. The major drawback of this method is antibody availability and specificity. Recently, DHS mapping has been conducted using an improved protocol, which includes the addition of circular carrier DNA, single cell DNase I seq (scDNase I-seq), requiring an input of between one and 1000 cells. This technology revealed highly expressed genic regions with multiple active histone marks displaying constitutive DNase I hypersensitive sites among different single cell analysis data. However, in scDNase I-seq the mappability of 1000 cells to the reference genome was 40%, and 2% at the single cell level [20].

Two other well-adapted methods for the identification of open chromatin and regulatory sites across the genome are FAIRE-seq (formaldehyde assisted isolation of regulatory element sequencing) and ATAC-seq (assay for transposase-accessible chromatin using sequencing) [21–23]. FAIRE-seq enriches the nucleosome-depleted DNA using formaldehyde fixation, sonication, and phenol/chloroform extraction of the non-protein-bound DNA. ATAC-seq uses a hyperactive Tn5 transposase which preferentially integrates its payload adaptor into accessible chromatin regions compared to less accessible, compact chromatin. Indeed, ATAC-seq maps of human CD4+ T cells from a proband on consecutive days demonstrated the feasibility of analyzing an individual’s epigenome on a timescale that is compatible with clinical decision-making [23]. However, ATAC-seq also generates non-specific amplification of non-nuclear DNA, such as the mitochondrial genome, accounting for ~50% of all reads. Both DNase-seq and FAIRE-seq can be applied to fixed cells but ATAC-seq works best on unfixed cell nuclei. The lack of a common metholodology for chromatin occupancy mapping in both unfixed (living) and fixed cells is a limitation of existing techniques.

Here we report a robust and sensitive method, nicking enzyme assisted sequencing (NicE-seq), for epigenetic profiling of the mammalian chromatin that can provide in-depth open versus closed chromatin sequence information from limited amounts of either native or fixed cells. We used NicE-seq to identify regions of transcription factor occupancy and compared these to DHSs identified by DNAse-seq and ATAC-seq identified sites. Finally, we studied the dynamic open chromatin sites (OCSs) of human colorectal cancer HCT116 cells before and after chemotherapeutic treatment with the DNA demethylating drug decitabine.

## Results

### Nicking enzyme-mediated tagging of the open chromatin

**Fig. 1:**
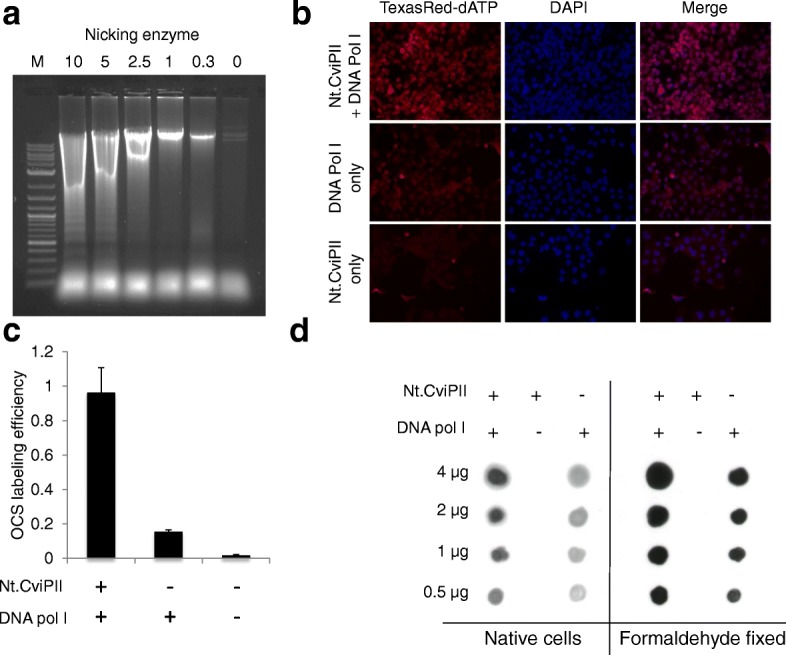
Nicking enzyme-mediated labeling of open chromatin. **a** Nicking of crosslinked chromatin using varying amounts of Nt.CviPII. A 1% agarose gel showing differential nicking of HCT116 genomic DNA based on amount of nicking enzyme (10 U, 5 U, 2.5 U, 1 U, 0.3 U, 0 U). *M* is a DNA molecular weight ladder. **b** Open chromatin labeling in fixed HeLa cells using dNTPs supplemented with TexasRed-dATP. *Top panel*: labeling reaction performed in the presence of Nt.CviPII and DNA polymerase I. *Middle panel*: labeling reaction performed in the presence of DNA polymerase I only. *Bottom panel*: labeling reaction performed in the absence of Nt.CviPII and DNA polymerase I. TexasRed-dATP was included in all reactions. DNA staining was performed using DAPI (*blue*) and TexasRed stain (*red*) represents labeled OCSs. Magenta stain (*Merge*) represents the colocalization. **c** Labeling efficiency of OCSs in all three assayed conditions. The *y-axis* represents the ratio of the intensity of the red pixels to the intensity of the blue pixels (*OCS labeling efficiency*). **d** Dot blot showing labeling of open chromatin by Nt.CviPII nicking enzyme in both native and formaldehyde-fixed HCT116 cells. The level of labeling was revealed using HRP-conjugated goat anti-biotin antibody

We isolated nuclei from colorectal cancer HCT116 cells and incubated the sample with the nicking enzyme Nt.CviPII, which nicks human genomic DNA with sequence specificity CCD (D = A/G/T). We hypothesized that Nt.CviPII would be able to travel across the nuclear membrane and nick open chromatin regions inside the nucleus. We used formaldehyde-fixed cell nuclei in buffer containing various concentrations of Nt.CviPII for a fixed time, extracted DNA, and analyzed the product on a 1% agarose gel. When sufficient enzyme was added, high molecular weight genomic DNA was digested and nucleosome banding patterns were observed, confirming the activity of Nt.CviPII on chromatin inside the nucleus. To avoid false positives in open chromatin nicking and to facilitate random and optimal nicking by the enzyme, we chose a median concentration of 2.5 unit of Nt.CviPII/μg of chromatin for all downstream experiments (Fig. 1a). The nicked open chromatin regions in fixed HeLa cells were filled-in using *Escherichia coli* DNA polymerase I plus dNTP mix supplemented with TexasRed-dATP to demonstrate the labeling of OCSs. The cells were scored for TexasRed-dATP incorporation and compared to DAPI for open chromatin index (OCI) measurement. Indeed, the cells without Nt.CviPII and *E. coli* DNA polymerase I (control) displayed no TexasRed signal (Fig. 1b, c), although a small amount of signal was observed when *E. coli* DNA polymerase I was used alone, suggesting endogenous nicks were present in the nucleus (Fig. 1b, c). In the open chromatin sequencing experiment, we used biotin-14-dATP and biotin-16-dCTP to generate biotin-tagged open chromatin regions. To validate incorporation of biotin in the chromatin, DNA was extracted, dot blotted, and probed with anti-biotin antibody (Fig. 1d). Thus, both native chromatin and formaldehyde-fixed chromatin were efficiently labeled with biotin and the labeling reaction could be visualized in the nucleus, suggesting that the nicking enzyme and *E. coli* DNA polymerase I were able to access the open chromatin.

### Open chromatin mapping with 250 cells

**Fig. 2:**
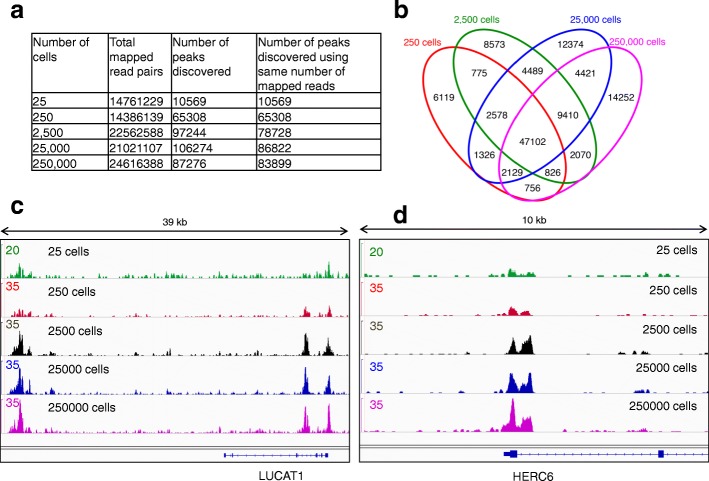
NicE-seq using 25–250,000 cells for open chromatin profiling. **a** The number of total mapped reads and the number of peaks identified before and after normalizing the total mapped reads to the level of 250 cells. **b** A Venn diagram showing the overlap between the OCSs identified from 250 to 250,000 cells. **c** A screenshot of the IGV browser showing the alignment of identified OCSs from 25 to 250,000 cells in a 39-kb window. **d** A screenshot of the IGV browser showing the alignment of identified OCSs from 25 to 250,000 cells in a 10-kb window

The genomic DNA from the biotin labeling reaction was purified, fragmented, and captured using Streptavidin beads for library construction. Streptavidin-captured DNA from putative open chromatin regions was used for high-throughput sequencing. The sequence information was analyzed by various peak-calling packages, such as MACS2, SICER, ZINBA, and PeaKDEcK, and about half of the peaks were common to all methods (Additional file 1: Figure S1). We subsequently used MACS2, a well established and routinely used peak calling package. To determine the robustness of NicE-seq, we also used different amounts of fixed cells ranging from 25 to 250,000. The number of observed peaks cells did not drop significantly when using between 2500 and 250,000 cells, but a 20% decrease occurred with 250 cells compared to 2500 (Fig. 2a). Furthermore, the NicE-seq library from 25 cells identified 10,569 peaks, with 8660 peaks overlapping with that from 25,000 cells (Fig. 2a; Additional file 1: Figure S2). The amount of overlapping peaks in libraries made using 250 to 250,000 cells was in the range 55–72%, suggesting good correlation (Fig. 2b). Considering both the peak numbers and overlaps, the lower limit for open chromatin mapping using NicE-seq under these conditions is 250 cells. We also examined if Nt.CviPII recognition sequence density has any influence on open chromatin enrichment by plotting nicking site density versus log2 fold enrichment of tags and observed poor correlation in 100-bp genomic tiles (Additional file 1: Figure S3a). We found a similarly poor correlation between number of nicking sites and sequence tags in the open chromatin peaks (Additional file 1: Figure S3b). These results suggest that the frequency of nicking sites has no bearing on open chromatin enrichment, but that the accessibility of the open chromatin to the nicking enzyme is the major determinant of tag reads (Additional file 1: Figure S3). Therefore, we termed the identified sequences/peaks as open chromatin sites (OCSs). We further visualized the OCSs between 25 and 25,000 cells using selected genic regions and observed consistent overlap in enrichment of sequence tags/peaks as seen in 39- and 10-kb windows (Fig. 2c, d), confirming the versatility of NicE-seq.

### NicE-seq identifies unique and divergent peaks on native or fixed chromatin

**Fig. 3:**
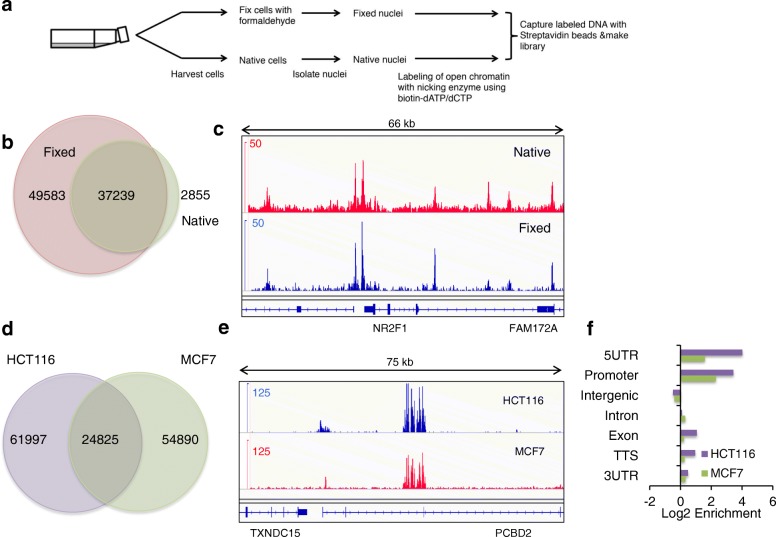
NicE-seq and validation of NicE-seq in fixed and native cells. **a** The different steps involved in labeling and capture of open chromatin sites using NicE-seq. **b** A Venn diagram showing the overlap between the OCSs identified using fixed and native HCT116 cells. **c** A screenshot of the IGV browser showing the overlap between OCSs identified in native (*top panel* in *red*) and fixed (*bottom panel* in *blue*) HCT116 cells in a 66-kb window. **d** A Venn diagram showing overlap between OCSs identified from HCT116 and MCF7 cell lines. **e** Snapshot of IGV browser showing overlap of OCS peaks (*top panel* in *blue* for HCT116 and *bottom panel* in *red* for MCF7) in a 75-kb window. **f** The log_2_ fold change of OCS peaks in different genomic regions for HCT116 (*purple*) and MCF7 (*green*) cells

To determine if the open chromatin configurations are preserved between native and formaldehyde-fixed cells, we performed NicE-seq of both HCT116 fixed and native cells (Fig. 3a). To our surprise the native cell chromatin samples displayed half the number of OCSs compared to the fixed cells (Fig. 3b). Most of the open chromatin peaks in native cells were a subset of those in fixed cells, suggesting native cell chromatin structure is dynamic and may prevent efficient incorporation of biotinylated dNTP (Fig. 3b, c). However, a comparison of the sequence read peaks from both confirmed that some peaks or OCSs were indeed relatively static and common, while others were dynamic (Fig. 3c; Additional file 1: Figure S4).

We also performed NicE-seq on MCF7 (breast carcinoma) cells and obtained 79,000 OCSs, compared to 87,000 for HCT116 cells. Further comparison between the OCSs of MCF7 and HCT116 cells showed an overlap of 24,000 peaks, suggesting the presence of both common and unique OCSs specific to the two cell types (Fig. 3d). A genome browser track also demonstrated both constitutive as well as unique OCSs in MCF7 cells (Fig. 3e; Additional file 1: Figure S5).

### NicE-seq and distribution of open chromatin marks in human cells

To further our understanding of the distribution of open chromatin in the genome, we annotated the genomic features of open chromatin regions identified by NicE-seq. The open chromatin peaks were mostly concentrated at gene promoters, specifically transcription start sites. In the genic region both introns and coding exons displayed varying degrees of open chromatin status (Fig. 3f).

**Fig. 4:**
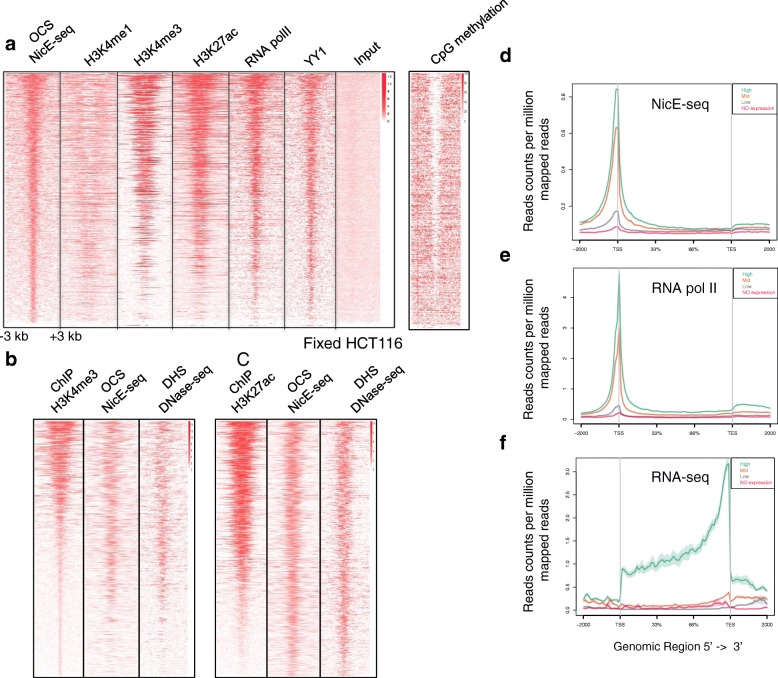
Validation of open chromatin sites identified by NicE-seq. **a** A heat map showing the correlation of NicE-seq peaks in a ±3-kb window with occupancy of H3K4me1, H3K4me3, H3K27ac, RNA pol II, and YY1 across ChIP-seq data from ENCODE for HCT116 cells. Input was used to show lack of enrichment. Plotting a heat map using whole genome bisulfite sequencing data for HCT116 cells showed an inverse correlation between OCSs and CpG methylation. **b** A heat map showing the correlation of ChIP H3K4me3 peaks in a ±3-kb window with NicE-seq and DNase-seq data sets. **c** A heat map showing the correlation of ChIP H3K27ac peaks in a ±3-kb window with NicE-seq and DNase-seq data sets. **d** Metagene plot showing the distribution of sequencing tag densities for high (*turquoise*), medium (*orange*), low (*purple*), and no expression (*pink*) genes (based on the expression level in the RNA-seq data set) in the NicE-seq experiment in a 2-kb window upstream of the transcription start site (TSS) and downstream of the transcription termination site (TTS). **e** Metagene plot showing the distribution of sequencing tag densities for high (*turquoise*), medium (*orange*), low (*purple*), and no expression (*pink*) genes (based on the expression level in RNA-seq data set) in the ChIP RNA pol II experiment in a 2-kb window upstream of the TSS and downstream of the TTS. **f** Metagene plot showing the distribution of sequencing tag densities for high (*turquoise*), medium (*orange*), low (*purple*), and no expression (*pink*) genes (based on the expression level in the RNA-seq data set) in a 2-kb window upstream of the TSS and downstream of the TTS in the RNA-seq data set

ENCODE datasets for various active chromatin marks and DNA-binding protein factors were investigated using OCSs identified by NicE-seq for further validation of the technique. We determined the distribution of tag densities for various ChIP-seq experiments (H3K4me1, H3K4me3, H3K27ac, RNA pol II, and YY1) in a ±3-kb window around the OCSs identified by NicE-seq and generated heat maps. NicE-seq heat maps positively correlated with RNA pol II binding, suggesting that most of the OCSs may be transcriptionally active. Furthermore, the signature transcriptional activation marks H3K27ac and H3K4me3 displayed strong enrichment around OCSs in the heat map. Also, H3K4me1, which is mainly enriched in the enhancer regions, was more depleted at the OCS peaks and showed a bimodal distribution around OCSs (Fig. 4a). Interestingly, YY1, a ubiquitously distributed transcription factor belonging to the GLI-Kruppel class of zinc finger protein that is involved in repression and activation of a diverse number of promoters, displayed a pattern similar to that defined by NicE-seq. As expected, the heat map for CpG methylation status inversely mirrored the NicE-seq open chromatin configuration (Fig. 4a). This provides strong evidence for validating OCSs identified by NicE-seq as truly open and being actively transcribed. We also looked for enrichment of ChIP-seq peaks for H3K4me3 and H3K27ac in both NicE-seq and DNase-seq experiments and observed strong correlation. This clearly demonstrates that transcriptionally active histone marks are correlated with open chromatin regions (Fig. 4b, c).

### Open chromatin and transcription

Transcription of mammalian genes is regulated by promoter elements and transcription factors. Transcription start sites (TSS) are speculated to be devoid of nucleosomes for transcription factor accessibility and have characteristic histone marks. NicE-seq tags were highly enriched at the TSS region of genes in HCT116 cells (Fig. 4d). We analyzed the ENCODE data set for MNase-seq and observed nucleosome depletion at the TSS corresponding to the open chromatin (Additional file 1: Figure S6a). Correlating with the open chromatin and lack of nucleosomes, this region also displayed high occupancy of RNA pol II, suggesting transcriptional activation (Fig. 4e). Indeed, the transcriptional activation marks H3K4me3 and H3K27ac are partially depleted at TSSs, due to a lack of nucleosomes, but are highly occupied at both distal and proximal ends (Additional file 1: Figure S6b, c). However, H3K4me1 was highly depleted at TSSs and was prominent at upstream enhancer and downstream regions (Additional file 1: Figure S6d). Further analysis of TSSs using NicE-seq data suggested that the rate of gene expression mirrored the degree of open chromatin conformation, i.e., more highly expressed genes contain highly open chromatin at the TSS and this is occupied by RNA pol II (Fig. 4e, f; Additional file 1: Figure S7). Highly open chromatin with RNA pol II occupancy displayed a high degree of RNA transcription.

### NicE-seq and DNase-seq identify an overlapping as well as unique set of open chromatin sites in human cells

**Fig. 5:**
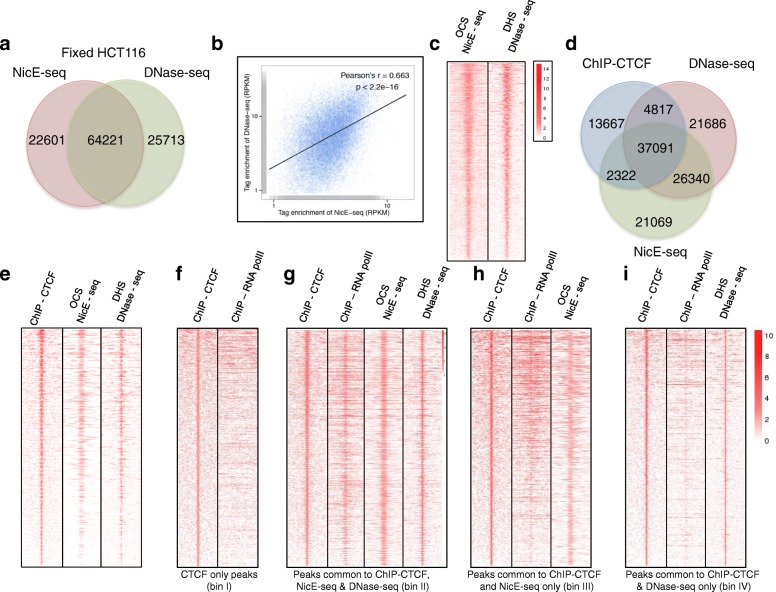
Comparison of OCSs and DHSs in HCT116 cells and correlation of CTCF binding with open chromatin regions. **a** Venn diagram showing the overlap between the OCSs and DHSs identified using NicE-seq and DNase-seq (ENCODE) for fixed HCT116 cells. **b** Correlation of tag enrichment (RPKM) for peaks common to NicE-seq and DNase-seq was determined using the Pearson’s linear correlation method for HCT116 cells. A linear regression line was also plotted. **c** A heat map showing the distribution of common peaks between NicE-seq and DNase-seq in a ±3-kb window for HCT116 cells. **d** A Venn diagram showing the overlap between ChIP-CTCF peaks (ENCODE), OCSs, and DHSs identified using NicE-seq and DNase-seq (ENCODE). **e** A heat map representing the distribution of ChIP-CTCF peaks in a ±3-kb window across ChIP-CTCF, NicE-seq, and DNase-seq data sets reveals similar patterns for enrichment of tag densities. **f** A heat map showing the correlation of peaks unique to ChIP-CTCF in a ±3-kb window with occupancy of RNA pol II revealed no enrichment for RNA pol II, suggesting the region is transcriptionally inactive. **g** A heat map showing the correlation of peaks common to ChIP-CTCF, NicE-seq, and DNase-seq data sets in a ±3-kb window with occupancy of RNA pol II revealed significant enrichment for RNA pol II, suggesting the region is transcriptionally active. **h** A heat map showing the correlation of peaks common to ChIP-CTCF and NicE-seq data sets in a ±3-kb window with occupancy of RNA pol II revealed enrichment for RNA pol II, suggesting the region is transcriptionally active. **i** A heat map showing the correlation of peaks common to ChIP-CTCF and DNase-seq data sets in a ±3-kb window with occupancy of RNA pol II revealed low enrichment for RNA pol II

DNase-seq and NicE-seq identify regions of chromatin devoid of nucleosomes and other DNA-binding proteins. To quantify the level of overlap between these assays, we identified the peaks common to both techniques. ENCODE data for DNase-seq analysis of HCT116 cells was used to call peaks as described in “Methods”. Among 86,000 and 90,000 peaks identified by both NicE-seq and DNase-seq, respectively, 75% overlapped and ~25% were unique (Fig. 5a). Comparison of genome browser track profiles for OCSs and DHSs from both NicE-seq and DNase-seq also displayed common peaks (Additional file 1: Figure S8a). We considered the overlapping sites from both DNase-seq and NicE-seq experiments to represent high confidence open chromatin sites. The Pearson’s correlation coefficient for the tag density of common peaks between DNase-seq and NicE-seq was found to be 0.66 with a highly significant *p* value of 2.2e^−16^. This suggested a high level of correlation between the two methods (Fig. 5b). Furthermore, a heat map showing the distribution of tag densities for DNase-seq and NicE-seq in a ±3-kb window around the common peaks displayed similar enrichment patterns (Fig. 5c). Annotation of peaks unique to either NicE-seq or DNase-seq and common both showed similar enrichment across different genomic regions (Additional file 1: Figure S8b). Furthermore, using NicE-seq and ENCODE DNase-seq data for the MCF7 cell line, we identified 79,000 and 133,000 peaks for NicE-seq and DNase-seq, respectively (Additional file 1: Figure S9a). Of these, 43,000 peaks overlapped with a Pearson’s correlation coefficient of 0.746 (*p* < 2.2 e^−16^), exhibiting strong correlation between OCSs and DHSs as seen in the heatmap (Additional file 1: Figure S9b, c). The broader dynamic range of DNase-seq may be due to non-specific nicking of the genome compared to sequence-specific nicking mediated by the nicking enzyme Nt.CviPII (Additional file 1: Figure S9b).

To correlate the transcriptional status, we analyzed the distribution of the active chromatin marks H3K4me3 and H3K27ac in peaks common to NicE-seq and DNase-seq, unique to NicE-seq, and unique to DNase-seq. Indeed, common peaks displayed high enrichment of H3K4me3 and H3K27ac marks corresponding to transcriptional activation (Additional file 1: Figure S10a). A similar enrichment profile for H3K4me3 was observed for unique NicE-seq peaks. However, H3K27ac levels remained consistent with unique NicE-seq peaks compared to slightly elevated enrichment of DNase-seq peaks (Additional file 1: Figure S10b, c), suggesting differential identification of open chromatin marks using these enzymes. Also, the mean FPKM value for peaks unique to DNase-seq was lower compared to peaks unique to NicE-seq and peaks common to both NicE-seq and DNase-seq (Additional file 1: Figure S10d).

### NicE-seq offers higher specificity for OCSs compared to DHSs from DNase-seq in human cells

CTCF binds strands of DNA together to form chromatin loops, and also anchors DNA to nuclear lamina. It defines the boundaries between active and heterochromatic DNA and is shown to be a gene repressor. CTCF recognizes and binds the CCCTC sequence motif in DNA, which is a subset of the trinucleotide CCD recognition sequence of Nt-CviPII. We hypothesized that any open chromatin region containing a CTCF binding site will be specifically targeted by Nt-CviPII and will be displayed positively in NicE-seq. To investigate the relationship between CTCF-bound regions and chromatin accessibility, we examined the distribution of peaks between NicE-seq, DNase-seq, and ChIP-CTCF experiments. Interestingly, 64% of ChIP-CTCF peaks were common to all three experiments, suggesting that these regions could in fact be open. Further, 68 and 72.3% of ChIP-CTCF peaks were associated with NicE-seq OCSs and DNase-seq DHSs, respectively (Fig. 5d). Also, we identified CTCF-bound peaks unique to OCSs and DHSs to comprise 4 and 8% of the total ChIP-CTCF peaks, respectively (Fig. 5d). Similar results were obtained for two other transcription factors, Max and Sp1 (Additional file 1: Figures S11 and S12).

CTCF has been shown to promote gene expression and CTCF-bound OCSs/DHSs may belong to the class of genes that are transcriptionally active. To further elucidate the transcriptional status of ChIP-CTCF peaks in the context of OCSs and DHSs, we analyzed the distribution of CTCF peaks in the RNA pol II ENCODE data set by binning the peaks four ways. Peaks in bin I (24% of total ChIP-CTCF peaks) were unique to CTCF and showed no enrichment for RNA pol II occupancy, suggesting binding of CTCF is associated with intergenic or closed chromatin status (Fig. 5f). We further binned the remaining 76% of ChIP-CTCF peaks into those common to ChIP-CTCF, NicE-seq, and DNase-seq (bin II), common to ChIP-CTCF and NicE-seq (bin III), and common to ChIP-CTCF and DNase-seq (bin IV) and analyzed RNA pol II occupancy. As expected, peaks from bin II displayed a strong correlation with RNA pol II occupancy (Fig. 5g). However, peaks from bin III (Fig. 5h) displayed a higher correlation with RNA pol II occupancy than those in bin IV (Fig. 5i), suggesting differential transcriptional status.

For validation of transcriptional status, we analyzed the distribution of the active chromatin marks H3K4me3 and H3K27ac in peaks common to ChIP, NicE-seq, and DNase-seq (bin II), peaks common to ChIP and NicE-seq (bin III), and peaks common to ChIP and DNase-seq (bin IV). Indeed, peaks from bin II displayed high enrichment of H3K4me3 and H3K27ac marks, corresponding to transcriptional activation (Additional file 1: Figure S13a). A similar enrichment profile was observed for bin III, but not for bin IV (Additional file 1: Figure S13b, c). This was also evident in ChIP fragment depth analysis. When common OCS and DHS sequences were extracted and plotted with ChIP-seq H3K4me3 and H3K27ac reads, about a quarter of peaks from the heat map were devoid of H3K4me3 or H3K27ac, suggesting the possibility of other open chromatin marks that are yet to be discovered or open chromatin regions that are structural to the nuclear genome.

Based on the lack of enrichment for H3K4me3 and H3K27ac marks, we believe that peaks in bin IV are not truly open. However, strong enrichment was observed in DNase-seq for these peaks (Additional file 1: Figure S13c). Peaks in bin IV amount to 12% of all the DHS peaks associated with CTCF bound peaks. However, OCSs identified by NicE-seq and bound by CTCF are truly open, as evidenced by enrichment of active chromatin marks (Additional file 1: Figure S13b). Taken together, we suggest that some of the DHS regions identified could be false positives. Further, FPKM values for ChIP-CTCF peaks common to NicE-seq and DNase-seq (bin II) and NicE-seq alone (bin III) showed good agreement, although peaks from DNase-seq alone (bin IV) displayed FPKM values that were significantly less than those for the former two (Additional file 1: Figure S13d). We also correlated the DHSs and OCSs with transcription based on corresponding common and unique peaks for Max and Sp1. In both cases the unique peaks of NicE-seq (bin III) displayed higher transcriptional activity compared to the unique DHS peaks (bin IV). Interestingly, the number of unique DHS peaks (bin IV) was 1.5 times higher when compared to unique OCS peaks (bin III) and yet these peaks displayed lower FPKM values, suggesting the presence of false positives in DHS peaks (Additional file 1: Figure S11b and S12b).

### NicE-seq and ATAC-seq identify an overlapping as well as unique set of open chromatin sites in human cells

Before comparing NicE-seq to ATAC-seq, we compared open chromatin regions of GM12878 cells using DNase-seq and ATAC-seq datasets. Open chromatin peaks identified by ATAC-seq (99,885 peaks) and DNase-seq (118,157 peaks) for GM12878 cells displayed an overlap of 57,697 peaks (48% of DNase-seq peaks), suggesting half of the open chromatin regions are method specific (Additional file 1: Figure S14a). When we compared the open chromatin peaks identified by ATAC-seq between two different cell lines, GM12878 cells (99,885 peaks) and primary human neonatal keratinocytes (93,313 peaks), the overlap was reduced to 29,294 peaks (29% of GM12878 peaks), suggesting a high level of variation in open chromatin structure across the two different cell lines (Additional file 1: Figure S14b).

**Fig. 6:**
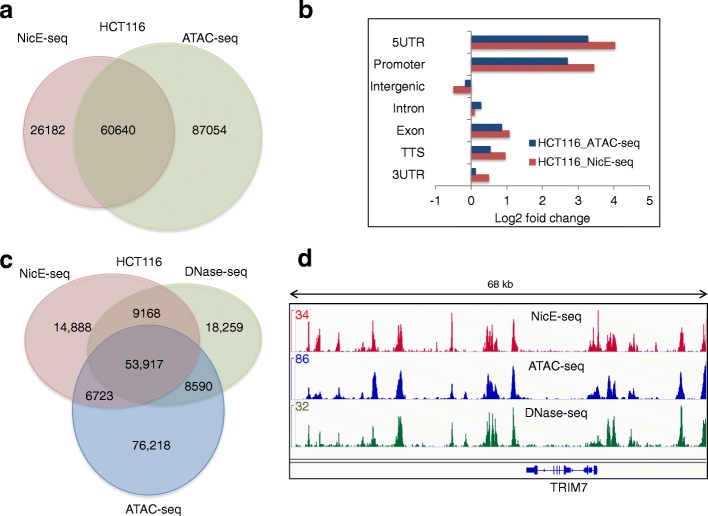
Comparison of open chromatin peaks identified by NicE-seq and ATAC-seq reveals both common and unique peaks. **a** A Venn diagram showing the overlap between NicE-seq and ATAC-seq for HCT116 cells. **b** The log_2_ fold change of open chromatin peaks in different genomic regions for NicE-seq (*red*) and ATAC-seq (*blue*) in HCT116 cells. **c** A Venn diagram showing the overlap between the open chromatin peaks identified by NicE-seq, ATAC-seq, and DNase-seq for HCT116 cells. **d** Snapshot of the IGV browser showing the overlap of OCS peaks (*top panel* in *red* for NicE-seq, *middle panel* in *blue* for ATAC-seq, and *bottom panel* in *green* for DNase-seq for HCT116 cells) in a 68-kb window

Finally we compared the open chromatin peaks identified by NicE-seq on HCT116 cells with ATAC-seq on GM12878 and HCT116 cells. ATAC-seq identified 99,885 and 147,694 open chromatin peaks in GM12878 and HCT116 cells versus 86,822 open chromatin peaks identified by NicE-seq in HCT116 cells. A significant number of OCS—amounting to 37,547 peaks (43% of NicE-seq peaks for GM12878 cells) and 60,640 peaks (70% of NicE-seq peaks for HCT116 cells)—overlapped between ATAC-seq and NicE-seq by at least a single base (Fig. 6a; Additional file 1: Figure S14c). Interestingly, the log_2_ fold enrichment of peaks across different genomic features was similar in both cell line data sets (Fig. 6b; Additional file 1: Figure S15). Comparison between NicE-seq (HCT116 cells) and ATAC-seq and DNase-seq (GM12878 cells) resulted in 34% overlap in peaks, similar to the comparison between two unrelated cell lines (Additional file 1: Figure S14b, d). However, when data sets for all three methods in HCT116 cells were compared, we observed 64% of OCSs overlapping with ATAC-seq and DNase-seq peaks, suggesting very good agreement between all three methods (Fig. 6c). When the data were visualized using the IGV browser, peaks common to all data sets displayed similar enrichment (Fig. 6d; Additional file 1: Figure S14e), although sequencing depth was higher for ATAC-seq and DNase-seq with ~190 million for GM12878 cells, 100 million for HCT116 cells, and 39 million mapped reads compared to ~20 million mapped reads for NicE-seq. Therefore, with low background and high mapping efficiency, NicE-seq offers insights into the distribution of open chromatin at low sequencing depths.

### DNA hypomethylation of HCT116 genome correlates with OCSs

**Fig. 7:**
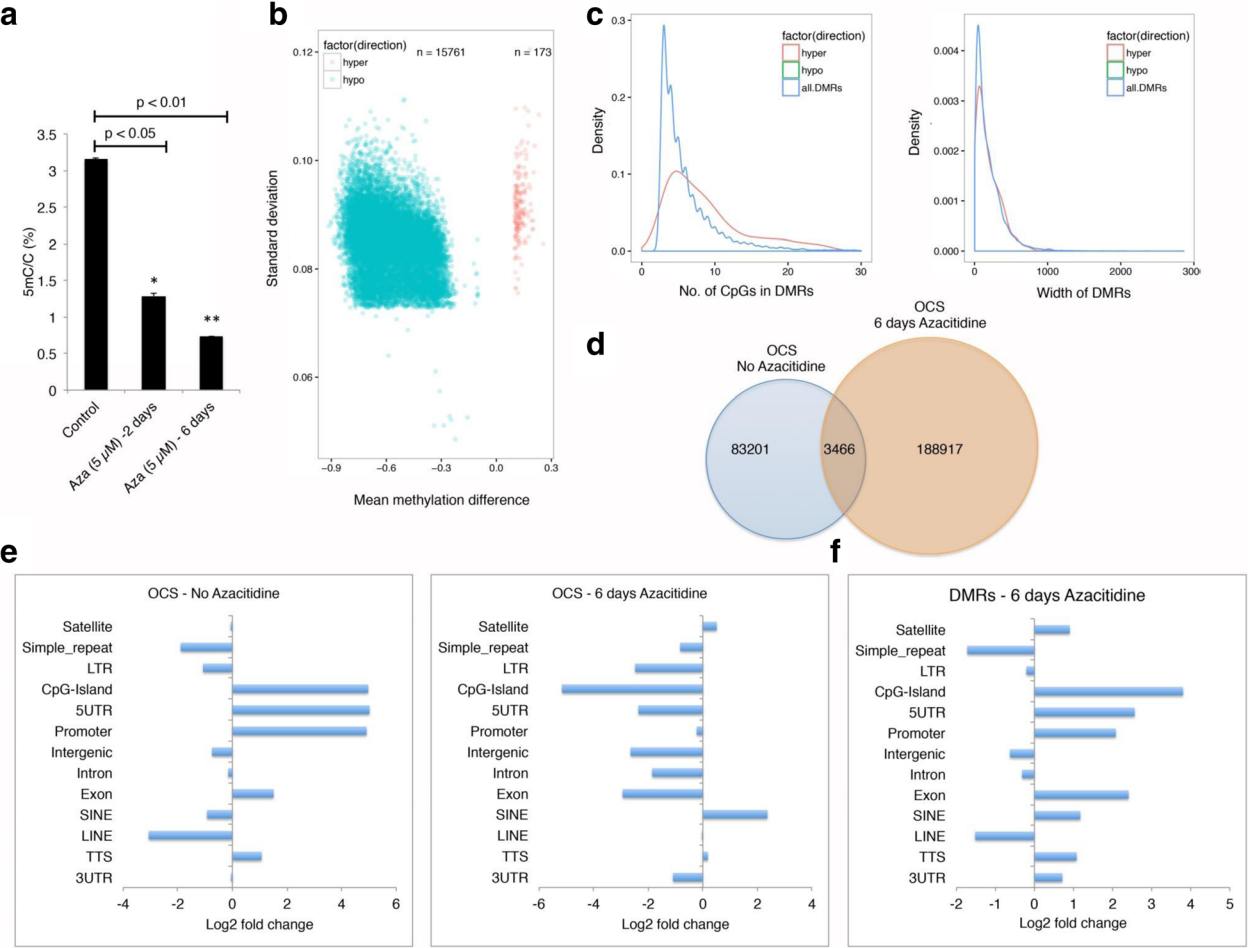
5-aza-2′-deoxycytidine affects nucleosome occupancy. **a** Liquid chromatography–mass spectrometry analysis showing loss of DNA methylation after treatment of HCT116 cells with 5 μM 5-aza-2′-deoxycytidine for 2 and 6 days. **b** Volcano plot showing the distribution of DMRs identified after 6 days of treatment. *Blue dots* represent hypomethylation (n = 15,761) and *red dots* represent hypermethylation (n = 173). **c** Density plots showing the distribution of number of CpGs in the DMRs and the width of DMRs identified in the study. **d** A Venn diagram showing the overlap between the OCSs identified with and without 6 days of 5 μM 5-aza-2′-deoxycytidine treatment. **e** The log_2_ fold change of OCS peaks in different genomic regions for samples treated with and without 6 days of 5 μM 5-aza-2′-deoxycytidine treatment. **f** The log_2_ fold change of DMRs in different genomic regions of samples treated with and without 6 days of 5 μM 5-aza-2′-deoxycytidine treatment

The cancer genome is globally hypomethylated. A high degree of DNA hypomethylation is also noted in transcriptionally active tumor suppressor genes in cancer cells. The demethylating drug decitabine is used for cancer treatment (for myelodysplastic syndromes, a class of conditions where certain blood cells are dysfunctional, and acute myeloid leukemia). We hypothesized that genome demethylation would lead to open chromatin and transcriptional activation of previously silenced genes. To test our hypothesis, we treated HCT116 cells with decitabine and measured levels of genomic 5mC. As expected, we observed a gradual demethylation of the genome and by the sixth day of treatment 5mC was reduced by 75% (Fig. 7a). Mean methylation difference analysis demonstrated ~99% of differentially methylated region (DMRs) were indeed hypomethylated (Fig. 7b). Although most of the hypomethylated DMRs had less than ten CpGs, the majority had three CpGs with a mean DNA length distribution of ~200 bp (Fig. 7c; Additional file 1: Figure S16a–d). OCS analysis between the control and 6-day decitabine-treated cells displayed a small percentage of common OCSs. Indeed, the vast majority of control OCSs were not represented in decitabine-treated cells and ~189,000 new OCSs were identified, suggesting large-scale chromatin landscape changes accompanying hypomethylation of the genome (Fig. 7d). In the untreated control cells, the OCSs primarily resided in CpG islands, 5′ UTRs, promoters, exons, and transcription termination sites (TTSs), as expected. Once the genome was hypomethylated, the SINE and satellite chromatin were opened. Surprisingly, previous OCSs at CpG islands, 5′ UTRs, promoters, exons, and TTSs were underrepresented. This observation suggests these regions were open to begin with and hypomethylation led to them becoming more open and thus more accessible to the nicking enzyme, resulting in degradation of the distal accessible DNA in the chromatin and underrepresentation of these regions (Fig. 7e). Also, the percentage increase in OCS peaks after 6 days of decitabine treatment was evenly distributed across all chromosomes as a function of their size (Additional file 1: Figure S17). Comparisons of DMRs between control and decitabine-treated cells suggested that most of the methylation changes occurred on chromatin representing CpG islands, 5′ UTRs, promoters, exons, SINEs, satellites, 3′ UTRs, and TTS regions (Fig. 7f).

## Discussion

Genome-wide studies using DNase-seq, FAIRE-seq, and ATAC-seq have provided crucial clues to regions of transcriptional regulation. Humans have approximately 1700 to 1900 transcription factors, which bind different types of DNA elements, including promoters, enhancers, silencers, insulators, and locus control regions. By comparing data from ChIP-seq, microarray, and genome-wide studies to detect open chromatin, one would be able to decipher crucial clues about gene regulation, particularly transcription. Classically, DHSs according to cell type specificity have been classified as common or unique. In one study, 900,000 DHSs were identified in 29 cell lines, accounting for almost 8.3% of the human genome [13]. Categorizing these DHSs further revealed that cell type-specific and ubiquitous DHSs are present in a 1:2 ratio and account for about 30,000 total peaks. The percentage of cell type-specific DHSs was much smaller than that found in a previous study [24]. Shu et al. [13] concluded this discrepancy may be due to the particular cell type since DNase I has a propensity to nick DNA at phosphodiester bonds, irrespective of the sequence context. NicE-seq, DNase-seq, and ATAC-seq in HCT116 cells identified 85,000–145,000 open chromatin peaks. Overlapping of NicE-seq peaks with either DNase-seq or ATAC-seq peaks was over 70%, suggesting authentic identification of OCS.

We have compared the random nicking activity of DNase I with a sequence-specific nicking enzyme, Nt-CviPII, and its impact on open chromatin identification. Nt-CviPII recognizes the CCD trinucleotide sequence and these sites are densely dispersed throughout the human genome. We have also demonstrated that the frequency of nicking sites has no bearing on open chromatin enrichment; rather, accessibility to the open chromatin by the nicking enzyme is the major determinant of tag reads of OCSs. However, when DHS and OCS reads were compared for HCT116 cells, about a third from either method remain unique. Additional transcriptional and histone mark analysis and comparison of these unique sequence tags revealed that the OCS peaks are more enriched in H3K4me3 and H3K27ac, suggesting Nt-CviPII has a higher specificity than DNase I, resulting in more accurate open-chromatin region determination. Furthermore, OCSs between two different cancer-derived cell lines demonstrated cell type-specific and common open chromatin regions, suggesting conservation of OCSs in mammalian cells. Comparison between NicE-seq, ATAC-seq, and DNase-seq demonstrated both unique and common open chromatin regions. This may be attributed to sequence and structural bias of DNase I or Nt-CviPII or biased tagging of DNA by the transposase used in ATAC-seq. Therefore, for limited numbers of cells, both NicE-seq and ATAC-seq may be performed to negate sequence bias.

When we used NicE-seq to determine the impact of decitabine-mediated hypomethylation on OCSs, we observed large numbers of OCSs compared to the control genome. DNA methylation of the genome attracts methyl CpG binding proteins and associated transcriptional repressors to confer a heterochromatin structure. In the absence of a DNA methylation signature, the heterochromatin structure is destabilized [25] and presumably chromatin is more open. This observation is supported by an earlier indirect chromatin accessibility study using array hybridization technology and the DNA methyltransferase inhibitor 5-aza-2′-deoxycytidine (5-Aza-CdR) or the histone deacetylase inhibitor suberoylanilide hydroxamic acid [26]. In another study, localized DNA demethylation at the H19 imprinting control region (ICR) induced by 5-AzaCdR reduced IGF2 expression, increased H19 expression, increased CTCF and cohesin recruitment, and also changed histone modifications along with chromatin accessibility [27].

Therefore, NicE-seq is a straightforward method that can be performed on potentially any cell type from any species with a sequenced genome. The protocol described here works for cell numbers between 250 and 25,000. Below 250 or above 25,000 cells the dynamic range of the current NicE-seq protocol is compromised, resulting in a drop in the number of peaks. This could be overcome by optimizing the concentration of nicking enzymes, reaction conditions, and cell numbers. Furthermore, different cell types and cells with different chromatin compaction, such as mitotic (compact chromatin) versus replicating (relaxed chromatin), will offer challenges to nicking enzyme catalysis. This may result in under representation of OCS for compact chromatin regions. The sequence specificity of Nt.CviPII (CCD) may cause it to misidentify AT-rich open chromatin sequences and thus may not be suitable for AT-rich genome studies. However, NicE-seq has similar resolution to ATAC-seq and DNase-seq in the human genome and no prior knowledge is required with regards to histone modifications, transcription factor binding sites, gene annotation, or relative degree of sequence conservation between species. It can aid in the identification of the most active gene regulatory elements in order to understand the chromatin landscape during mammalian development programming and epigenetic drug discovery studies.

## Conclusions

We demonstrate that the sequence-specific nicking enzyme Nt.CviPII can be used to probe open chromatin landscapes in a variety of cancer cells. NicE-seq captures OCSs in both fixed and native cells and reveals the genomic location of these and transcription factor occupancy at single-nucleotide resolution, with as few as 25 cells. Using NicE-seq we have demonstrated that colorectal cancer cells treated with the anti-cancer drug decitabine displayed time-dependent accumulation of OCSs coinciding with hypomethylation of the genome, particularly in SINEs and satellite repetitive DNA. DMRs were more pronounced in promoters, 5′ UTRs, CpG islands, and exons. Therefore, NicE-seq offers a quick and reliable method for open chromatin analysis in biological and pharmacological studies. We envision that when NicE-seq is coupled with fluorescent-labeled dNTPs it will facilitate visualization of open chromatin in eukaryotic cells.

## Methods

### Cell culture and 5-aza-2′-deoxycytidine treatment

HCT116 cells were cultured in McCoy’s 5A media supplemented with 10% fetal bovine serum (FBS). Cells (25,000) were treated with 5 μM 5-aza-2′-deoxycytidine (decitabine) for 6 days [28]. Samples were harvested after 2 days of treatment and the medium was replaced and fresh 5-aza-2′-deoxycytidine was added for another 4 days. MCF7 and HeLa cells were cultured in DMEM plus 10% FBS.

### Open chromatin labeling

For library construction, we used HCT116 and MCF7 cells. Cells were cross-linked using 1% formaldehyde for 10 min at room temperature and quenched by using 125 mM glycine. Nuclei were isolated by incubating the cross-linked cells in cytosolic buffer (15 mM Tris-HCl pH 7.5, 5 mM MgCl_2_, 60 mM KCl, 0.5 mM DTT, 15 mM NaCl, 300 mM sucrose, and 1% NP40) for 10 min on ice with occasional agitation. Nuclei were precipitated by spinning at 1000 × *g*, 4 °C for 5 min and the supernatant was discarded. Open chromatin DNA was labeled with biotin by incubating the nuclei in the presence of 2.5 U of Nt.CviPII (NEB R0626S), 10 U of DNA polymerase I (M0209S) and 30 μM of each dNTP including 6 μM of biotin-14-dATP (Invitrogen, 19524-016) and 6 μM of biotin-16-dCTP (ChemCyte, CC-6003-1) in 200 μL of 1× NEB buffer 2. The labeling reaction was carried out at 37 °C in a thermo-mixer at 800 RPM for 2 h. We added 20 μL of 0.5 M EDTA and 2 μg of RNase A to the labeling reaction and incubated it at 37 °C for 0.5 h to stop the reaction and digest RNA. Finally DNA–protein cross-linking was reversed by adding 20 μL of proteinase K (NEB, P8107S) and 20 μL of 20% SDS to the reaction and incubating at 65 °C for overnight. Biotin-labeled genomic DNA was extracted using phenol chloroform. Open chromatin labeling in native cells was performed without the formaldehyde crosslinking step.

Open chromatin labeling for microscopy was performed by culturing HeLa cells in an eight-well Lab-Tek II chambered coverglass system (Nalge Nunc International, 155409). The cells were fixed using 1% paraformaldehyde for 10 min and then washed thrice with 1× PBS for 5 min followed by incubation with cytosolic buffer for 10 min. The nicking reaction mix was as described earlier but with biotinylated dATP and dCTP replaced by TexasRed-dATP (PerkinElmer, NEL471001EA) for 1 h followed by washing the cells with 1× PBS supplemented with 50 mM EDTA and 0.1% TritonX-100 (wash buffer) for 5 min. Nuclear staining was performed by 0.01% Hoechst stain for 5 min. Finally, cells were washed thrice with wash buffer for 10 min each and visualized using a Zeiss LSM880 confocal microscope with 20× objective.

### Quantification of labeling efficiency

Open chromatin labeling efficiency was analyzed by dot blot on genomic DNA and by imaging labeled cells. A serial dilution of genomic DNA was spotted onto positively charged nylon membrane (Roche, 11209299001) and cross-linked by UV. Membrane was blocked by 5% non-fat milk and probed using an HRP-conjugated goat anti-biotin antibody (1:2000 dilution, CST, 7075). Biotin signal was revealed using LumiGLO reagent (CST, 7003).

### NicE-seq library construction

Biotin-labeled genomic DNA from fixed (HCT116, MCF7, and decitabine-treated HCT116 cells) and native (HCT116 cells) reactions were sonicated into 150-bp fragments (Covaris) and 1 μg of DNA was end-repaired, dA-tailed, and ligated with NEB Illumina adaptor (E7370S). Without further purification, the ligation product was mixed with 50 μL of Streptavidin magnetic beads (Invitrogen 65001, blocked using 0.1% cold fish gelatin in 1× PBS overnight at 4 °C) in 1 mL of B&W buffer (10 mM Tris-HCl pH 8.0, 1 mM EDTA, 2 M NaCl). Biotin-labeled open chromatin DNA was captured by streptavidin magnetic beads at 4 °C for 2 h with end-over-end rotation. The beads were washed four times with B&W buffer plus 0.05% of Triton X-100 followed by one wash with TE plus Triton X-100. The beads were resuspended in 40 μL of nuclease-free water and 4 μL was used for library amplification using PCR (NEB, E7370S). Ten PCR cycles were usually sufficient to generate the amount of library DNA needed for sequencing.

For library construction using 250 cells, all procedures remained the same except for the addition of 10 μg of glycogen during genomic DNA extraction. In the case of the 25-cell experiments, 0.25 U of Nt.CviPII and 5 U of DNA polymerase I were used. The entire DNA was sonicated and 10 μL of streptavidin beads were added to capture the biotinylated DNA, which was used as template for library amplification. For input library preparation 1 μg of purified genomic DNA was used instead of the labeled chromatin.

### ATAC-seq library construction

ATAC-seq was performed on 25,000 HCT116 cells according to the previously described protocol [29] with the following modifications. After addition of lysis buffer the cell suspension was passed through a 27 G ½ needle six times to facilitate cell lysis. ATAC-seq library was size-selected for 250–500 bp and 75-bp paired-end sequenced on the NextSeq 500 Illumina platform.

### Read mapping and open chromatin peak calling

Adaptor and low quality sequences were trimmed from paired-end sequencing reads using Trim Galore (http://www.bioinformatics.babraham.ac.uk/projects/trim_galore/) with default settings. Sequencing reads were mapped to reference human genome hg19 with Bowtie2 [30]. Four different packages with different algorithms were used for calling open chromatin peaks. MACS2 was used with -f BAMPE, -g hs, --broad, --broad-cutoff 0.1, --bdg, --keep-dup 1, -m 5 50, --slocal 1000, --llocal 10000 [31], ZINBA was used with extension = 360, printFullOut = 1 and refinepeaks = 1 [32], PeaKDEck was used with -sig 0.0001 [33], and SICER was used with a window size of 200 bp, fragment size of 150 bp, gap size of 600 bp, and FDR of 0.01 [34]. In all cases input library sequences were supplied if required. The input library was generated using sonicated genomic DNA of normal HCT116 cells. Peaks called by four different packages were considered associated if they have at least one base pair overlapping. Venn diagrams were plotted based on overlapping analysis.

In order to compare NicE-seq data when using different numbers of input cells (25–250,000), we downsized the mapped reads from different experiments to 14.4 million mapped read pairs. Peaks were called using the same parameter with MACS2 as mentioned above.

### Association analysis of Nt.CviPII site density and open chromatin tag enrichment

To exclude the possibility that open chromatin DNA enrichment is solely caused by the occurrence of Nt.CviPII sites (CCD, D = A or G or T) in certain genomic regions but not the accessibility of chromatin by labeling enzymes, we did correlation analysis on CCD site density and open chromatin tag density in 100-bp genomic tiles or open chromatin peak regions called by MACS2. Hg19 was scanned for Nt.CviPII sites using an in-house Perl program and the genomic coordinates of Nt.CviPII sites were recorded. Nt.CviPII site density was expressed as sites per kilobase genomic sequence. Pearson linear correlation was implemented on the number of Nt.CviPII sites and the number of sequencing tags or Nt.CviPII site density and log2 fold enrichment of open chromatin tags in MACS2 peak regions. The Pearson product-moment correlation coefficient was used to measure the degree of correlation.

### Comparison of NicE-seq with other chromatin profiling methods

We compared NicE-seq, ATAC-seq with DNase I hypersensitivity sequencing (referred throughout as DNase-seq) for sensitivity and specificity. DNase-seq data was downloaded from ENCODE (wgEncodeUwDnaseHct116AlnRep1 and wgEncodeUwDnaseMcf7AlnRep1) [35]. ATAC-seq data for HCT116 was generated in-house, GM12878 and primary human neonatal keratinocytes were obtained from Gene Expression Omnibus GSE47753 and GSM1645708 respectively. Sequencing reads were mapped and open chromatin peaks were called using MACS2 with the same parameters as the NicE-seq experiment. Overlapped open chromatin peaks in both methods were identified using DiffBind package in R [36]. For a more detailed comparison between NicE-seq and DNase-seq, the RPKM values of common peaks were correlated using the Pearson linear correlation method. The tag density of common peaks was also plotted on a heatmap to reveal the fine alignment of NicE-seq and DNase-seq peaks in common genomic regions. Data sets for DNase-seq (HCT116), ATAC-seq (HCT116, GM12878) and NicE-seq (HCT116) was loaded in bedGraph format on IGV browser for visualization.

### Analysis of association between open chromatin, histone modifications, transcription factor binding, and CpG methylation status

Open chromatin peaks identified by NicE-seq were correlated with ChIP-seq targeted to H3K4me1, H3K4me3, H3K27ac, RNApolII, and YY1 identified in the HCT116 cell line (ENCODE project files wgEncodeEH002874, wgEncodeEH000949, wgEncodeEH002873, wgEncodeEH001627, and wgEncodeEH001671). The sequencing tag density of chromatin and transcription factor marks on open chromatin peaks was counted using HOMER [37], and heat maps were plotted using the pheatmap package in R [38]. For the association analysis of CpG methylation and open chromatin, whole genome bisulfite sequencing data of the HCT116 cell line was retrieved from GEO (accession GSM1465024), CpG sites were mapped to the ±3-kb region of open chromatin, and the methylation ratio of single CpG sites was plotted on a heat map.

For correlation between CTCF occupancy and chromatin status, peaks for the ChIP-CTCF data set from ENCODE (wgEncodeEH003220) were called using MACS2. Overlap between peaks identified by ChIP-CTCF, NicE-seq, and DNase-seq was determined by plotting a Venn diagram using DiffBind [39]. Further, ChIP-CTCF peaks were binned into four bins; bin I represented peaks unique to the ChIP-CTCF data set; bin II represented peaks common to all three data sets; bin III represented peaks common to the ChIP-CTCF and NicE-seq data sets; and bin IV represented peaks common to the ChIP-CTCF and DNase-seq data sets using bedops package [40]. Occupancy of RNA pol II for peaks in these four bins was determined by counting the sequencing tag density for RNA pol II using HOMER and plotted as heat maps using pheatmap. Similar heat maps were plotted for determining the enrichment of H3K4me3 and H3K27ac for bins II, III, and IV. ChIP fragment depth analysis was performed using HOMER for H3K4me3 and H3K27ac data sets. Peaks from ChIP-Max (wgEncodeHaibTfbsHct116MaxV0422111AlnRep1) and ChIP-SP1 (wgEncodeHaibTfbsHct116Sp1V0422111AlnRep1) ENCODE data sets were also analyzed by binning as mentioned above.

### Correlation of chromatin accessibility with gene expression

Based on the expression level, all of the genes discovered by RNA-seq were divided into four groups, high, medium, low, and no expression. RNA-seq data were from a previous study using the HCT116 cell line (GSM1661373, GSM1661374, GSM1661375) [41]. Sequencing tag density of NicE-seq and other ChIP-seq data across the gene body was calculated and plotted using the ngsplot package [42]. Box plot of FPKM values for peaks corresponding to bins II, III, and IV for ChIP-CTCF, ChIP-Max, and ChIP-SP1 was plotted using the ggplot2 R package [43].

### Liquid chromatography–mass spectrometry analysis for quantifying global 5mC level

Genomic DNA of control and decitabine treated (days 2 and 6) was digested with NEB nuclease cocktail for 1 h and nucleoside analysis was performed using liquid chromatography–mass spectrometry for quantification of 5mC levels [44].

### Genome-wide DNA methylation analysis

Genomic DNA (1 μg) isolated from HCT116 cells treated with 5 μM 5-aza-2′-deoxycytidine for 6 days or untreated control was sonicated into 150-bp fragments, bisulfite treated, library prepared, and sequenced on the Illumina NextSeq 500 platform with 150-bp paired-end reads for whole genome bisulfite sequencing analysis. Adaptor and low quality sequences (Phred score <20) were trimmed and reads were mapped to hg19 using Bismark with Bowtie2 [45]. CpG methylation levels were calculated with uniquely mapped reads using Bismark methylation extractor with the parameter -p --no_overlap and a minimum coverage of 3. Differential methylation analysis was carried out using the bsseq R package [46]. CpGs present in at least two replicates of each group were retained for downstream analysis. DMRs were identified containing a minimum of three CpGs and mean difference between the control and 5-aza-2′-deoxycytidine-treated samples of greater than 0.1 using the bsseq R package. DMRs were annotated by mapping the genomic coordinates to various genomic regions using the annotatePeaks.pl function of HOMER [37]. DMRs were visualized by plotting a volcano plot using the ggplot2 R package [43]. Density plots for number of CpGs in DMR and the lengths of DMRs were plotted using the bsseq R package.

## Additional file

Additional file 1: Supplementary Figures S1–17. (DOCX 1378 kb)

## Abbreviations

ATAC-seq: Assay for transposase-accessible chromatin with high throughput sequencing
ChIP: Chromatin-immunoprecipitation
DHS: DNase I hypersensitivity site
DMR: Differentially methylated region
DNase-seq: DNase I-based sequencing
FAIRE-seq: Formaldehyde assisted isolation of regulatory element sequencing
LINE: Long interspersed nuclear element
NicE-seq: Nicking enzyme accessible chromatin sequencing
OCS: Open chromatin site
scDNase I-seq: Single cell DNase I-seq
SINE: Short interspersed nuclear element
TSS: Transcription state site
TTS: transcription termination site
UTR: Untranslated region
