# Supplementary material for: NicE-seq: high resolution open chromatin profiling
Source: Genome Biol. 2017 Jun 28;18:122. doi: 10.1186/s13059-017-1247-6 (PMC5488340; doi:10.1186/s13059-017-1247-6)
Supplement: Additional file 1: — Supplementary Figures S1–17. (DOCX 1378 kb) [file 13059_2017_1247_MOESM1_ESM.docx]

**NicE-seq: high resolution open chromatin profiling**

**Additional file 1: Supplementary Figures**

**
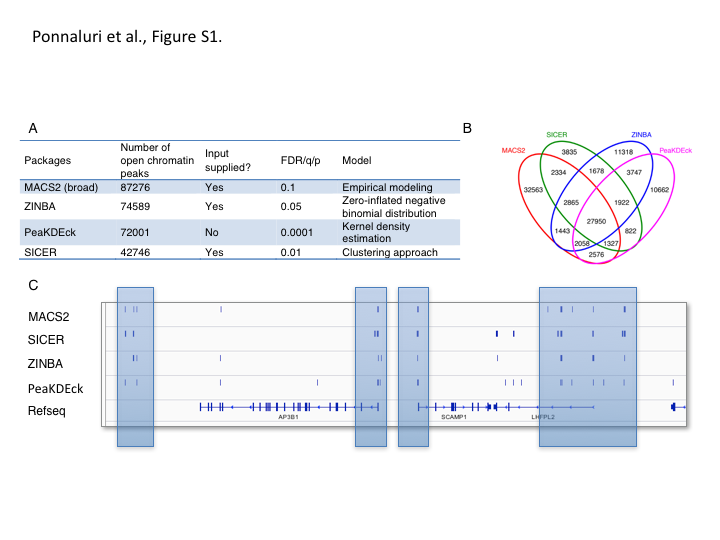
**

**Additional file1 Figure S1. Open chromatin sites analysis by different peak calling packages**

1. Table listing the number of OCS identified using different peak calling packages. Details about requirement of input and statistical model used for each peak caller are listed.
2. Venn diagram showing the overlap between OCS peaks identified using different peak calling packages.
3. Snapshot of an IGV browser showing distribution of OCS peaks called by different peak calling packages.

**
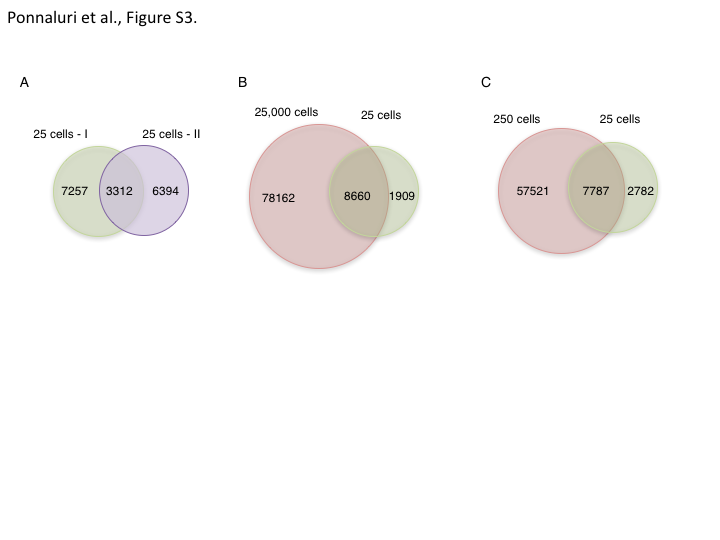
**

**Additional file1 Figure S2.** **NicE-seq identifies OCSs in as low as 25 cells.**

1. A Venn diagram showing the overlap of OCS identified between the two replicates of 25 cells using NicE-seq for HCT116 cells.
2. A Venn diagram showing the overlap of OCS identified between 25 and 25,000 cells using NicE-seq for HCT116 cells.
3. A Venn diagram showing the overlap of OCS identified between 25 and 250 cells using NicE-seq for HCT116 cells.

**
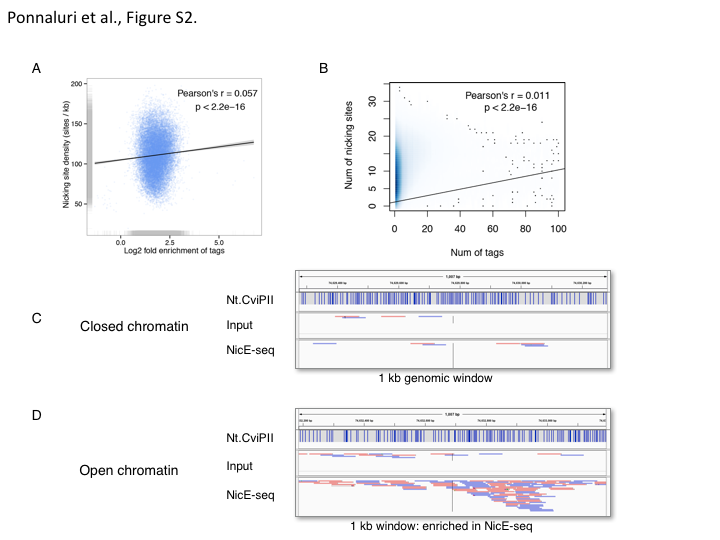
 Additional file1 Figure S3. Open chromatin enrichment is not highly associated with Nt.CviPII density in the genome**

1. Scatter plot showing no correlation between nicking site density and log_2_ fold enrichment of sequencing tags for NicE-seq using Pearson’s correlation test with r of 0.057 and p < 2.2 E^-16^.
2. Scatter plot showing no correlation between number of nicking sites and the number of sequencing tags obtained from NicE-seq using Pearson’s correlation test with r of 0.011 and p < 2.2 E^-16^.
3. Snapshot of an IGV browser showing Nt.CviPII nicking site distribution and distribution of sequencing reads for Input and NicE-seq samples in a 1 kb window. Lack of enrichment of reads in NicE-seq pane suggesting closed chromatin confirmation.
4. Snapshot of an IGV browser showing Nt.CviPII nicking site distribution and distribution of sequencing reads for Input and NicE-seq samples in a 1 kb window. Enrichment of reads in NicE-seq pane suggesting open chromatin confirmation.

**
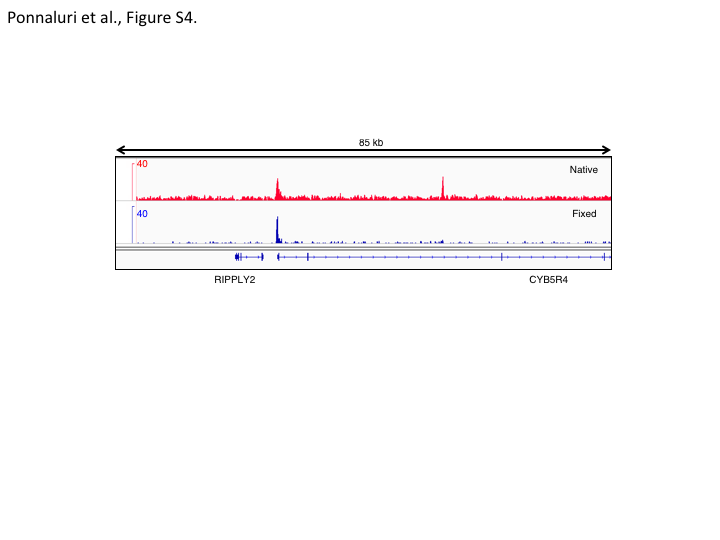
**

**Additional file1 Figure S4. NicE-seq identifies divergent peaks between native and fixed HCT116 cells.**

Snapshot of IGV browser showing OCS peaks identified by NicE-seq (top panel in red for native and bottom panel in blue for fixed) in a window of 75 kb.

**
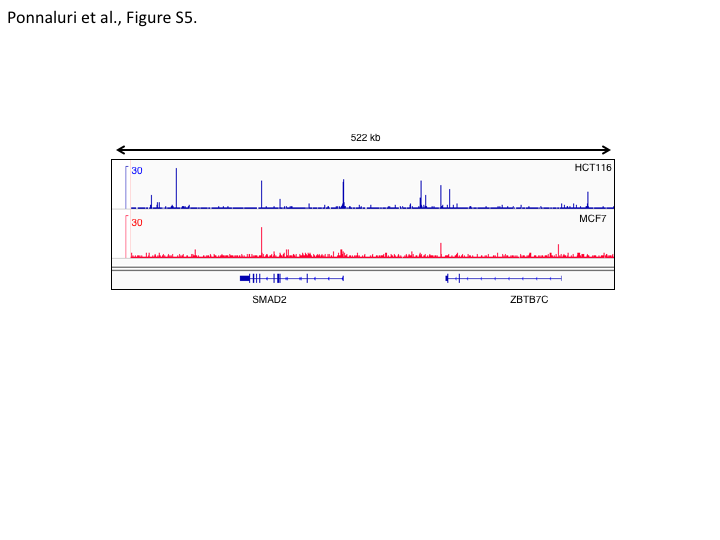
**

**Additional file1 Figure S5. NicE-seq identifies divergent peaks between HCT116 and MCF7 cells.**

Snapshot of IGV browser showing OCS peaks identified by NicE-seq (top panel in blue for HCT116 and bottom panel in red for MCF7) in a window of 75 kb.

**
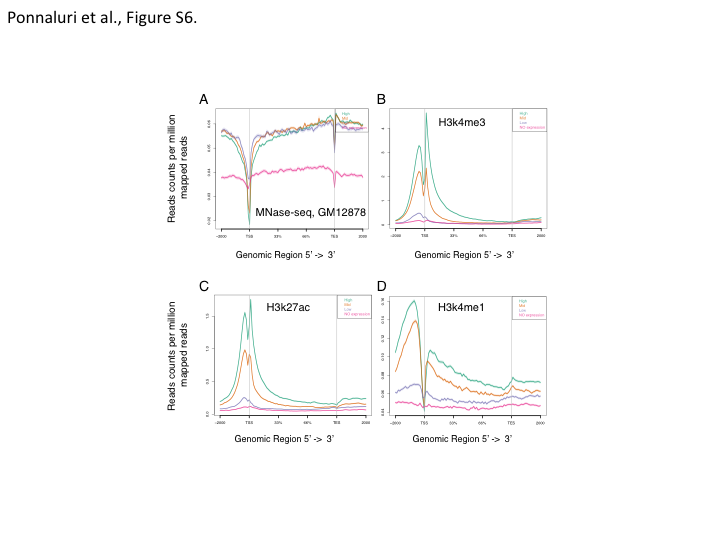
 Additional file1 Figure S6.** **Open chromatin signature on gene body based on nucleosome occupancy and histone modifications.**

Distribution of sequencing tag densities for high (turquoise), medium (orange), low (purple) and no expression (pink) genes in (A) MNase-seq (B) ChIP H3K4me3 (C) ChIP H3K27ac and (D) ChIP H3K4me1.

**
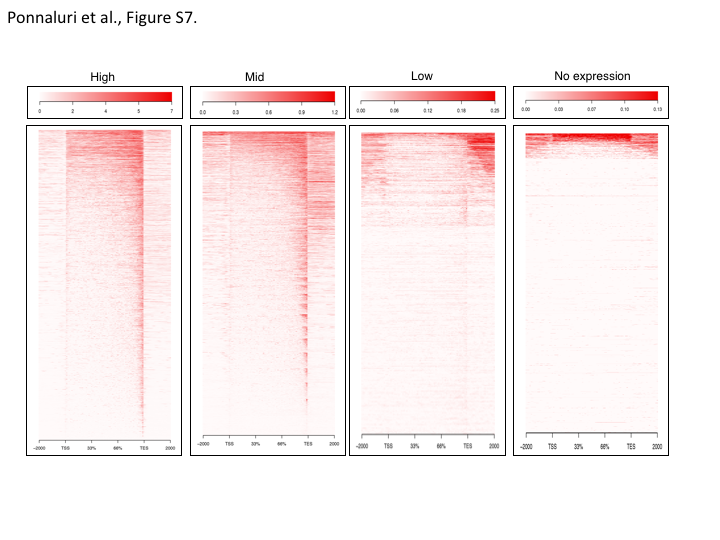
**

**Additional file1 Figure S7.** **Distribution of transcripts across gene body.**

Heatmap showing the distribution of sequencing tag densities for high, mid, low, and no expressing genes in ± 2 kb window around TSS and TES in RNA-seq data set.

**
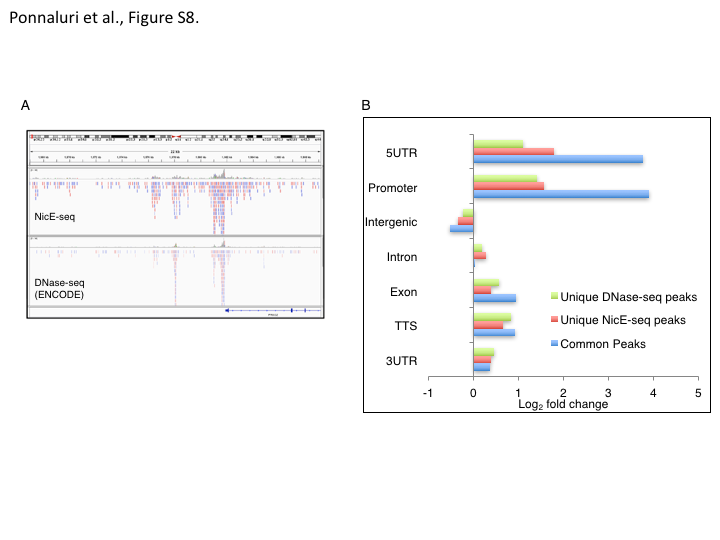
**

**Additional file1 Figure S8. Comparison between NicE-seq and DNase-seq peak distribution.**

1. A screenshot of IGV browser showing alignment of reads between NicE-seq, and DNase-seq for HCT116 cells in a window of 22 kb.
2. Bar graphs showing the log_2_ fold change of peaks common to NicE-seq and DNase-seq, unique NicE-seq peaks and unique DNase-seq peaks in different genomic regions.

**
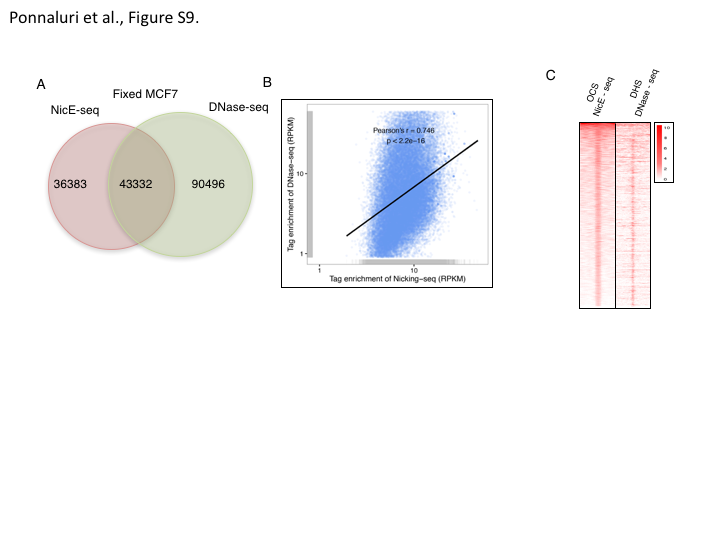
**

**Additional file1 Figure S9. Comparison of OCS and DHS in MCF7 cells.**

1. A Venn diagram showing the overlap between the OCSs and DHSs identified using NicE-seq and DNase-seq (ENCODE) for fixed MCF7 cells.
2. Correlation of tag enrichment (RPKM) for peaks common to NicE-seq and DNase-seq was determined using Pearson’s linear correlation method for MCF7 cells. A linear regression line was also plotted.
3. A heat map showing the distribution of common peaks between NicE-seq and DNase-seq in a ± 3 kb window for MCF7 cells.

**
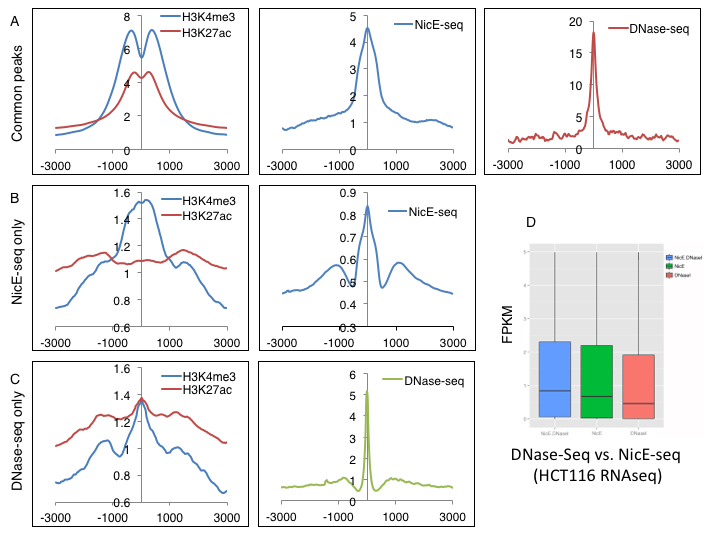
**

**Additional file1 Figure S10. ChIP fragment depth analysis of peaks from NicE-seq and DNase-seq for H3K4me3 and H3K27ac ENCODE data sets**

1. Left panel corresponds to distribution of tag density for peaks common to both NicE-seq and DNase-seq in H3K4me3 and H3K27ac data sets. Middle panel corresponds to distribution of tag density for OCS peaks in NicE-seq data set. Right panel corresponds to distribution of tag density for DHS peaks in DNase-seq data set.
2. Left panel corresponds to distribution of tag density for unique OCS peaks in H3K4me3 and H3K27ac data set. Right panel corresponds to distribution of tag density for unique OCS peaks in NicE-seq data set.
3. Left panel corresponds to distribution of tag density for unique DHS peaks in H3K4me3 and H3K27ac data set. Right panel corresponds to distribution of tag density for unique DHS peaks in DNase-seq data set.
4. Box plot showing the FPKM values for transcripts from peaks common to NicE-seq and DNase-seq, unique NicE-seq peaks and unique DNase-seq peaks from HCT116 RNAseq ENCODE data set.

**
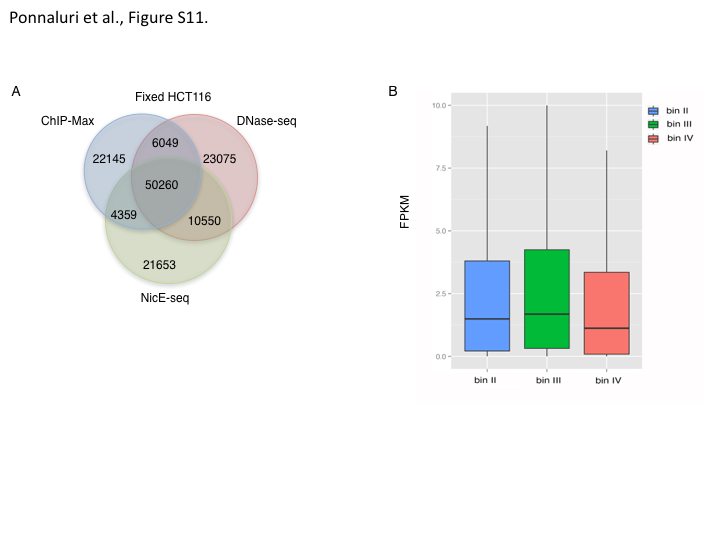
**

**Additional file1 Figure S11. Correlation of OCS and DHS peaks with ChIP-Max peaks for HCT116 cells.**

1. Venn diagram showing the overlap between OCS peaks (NicE-seq), DHS peaks (DNase-seq) and ChIP-Max peaks.
2. Box plot showing the FPKM values for transcripts corresponding to ChIP-Max peaks common with NicE-seq and DNase-seq (bin II), NicE-seq peaks (bin III), and DNase-seq peaks (bin IV) from HCT116 RNAseq ENCODE data set.

**
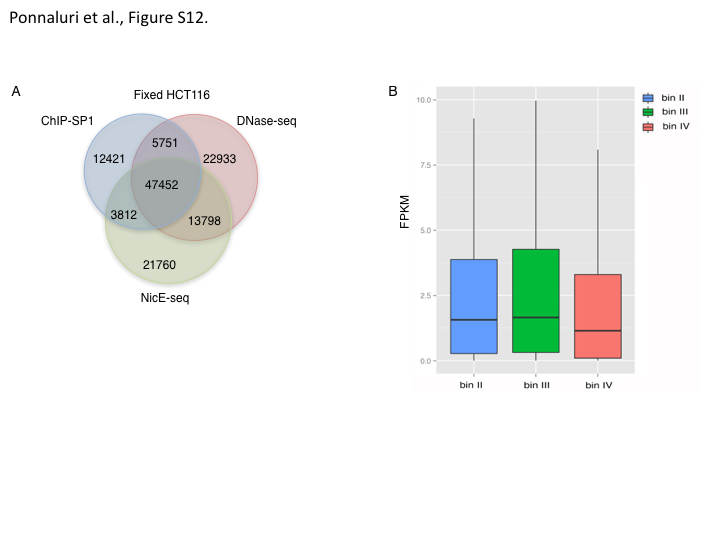
**

**Additional file1 Figure S12. Correlation of OCS and DHS peaks with ChIP-SP1 peaks for HCT116 cells.**

1. Venn diagram showing the overlap between OCS peaks (NicE-seq), DHS peaks (DNase-seq) and ChIP-SP1 peaks.
2. Box plot showing the FPKM values for transcripts corresponding to ChIP-SP1 peaks common with NicE-seq and DNase-seq (bin II), NicE-seq peaks (bin III), and DNase-seq peaks (bin IV) from HCT116 RNAseq ENCODE data set.

**
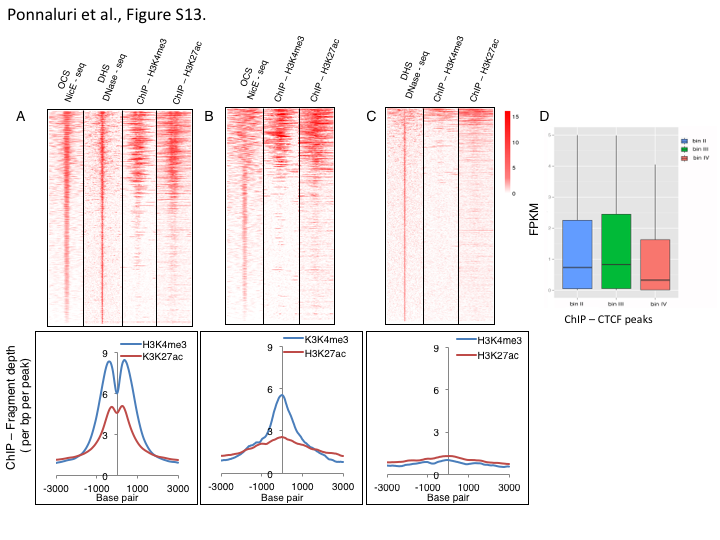
**

**Additional file1 Figure S13. NicE-seq offers higher specificity for OCS compared to DHS of DNase-seq.**

1. Top panel represents a heat map showing the correlation of peaks from bin II (peaks common to ChIP – CTCF, NicE-seq, and DNase-seq data sets) in a ± 3 kb window with occupancy of H3K4me3 and H3K27ac. Significant enrichment for both marks is observed suggesting the region to be transcriptionally active. Bottom panel shows the scatter plot of ChIP fragment depth for H3K4me3 and H3K27ac marks in a ± 3 kb window.
2. Top panel represents a heat map showing the correlation of peaks from bin III (peaks common to ChIP – CTCF and NicE-seq data sets) in a ± 3 kb window with occupancy of H3K4me3 and H3K27ac. Significant enrichment for both marks is observed suggesting the region to be transcriptionally active. Bottom panel shows the scatter plot of ChIP fragment depth for H3K4me3 and H3K27ac marks in a ± 3 kb window.
3. Top panel represents a heat map showing the correlation of peaks from bin IV (peaks common to ChIP – CTCF and DNase-seq data sets) in a ± 3 kb window with occupancy of H3K4me3 and H3K27ac. No significant enrichment for both marks is observed suggesting the region to be transcriptionally inactive. Bottom panel shows the scatter plot of ChIP fragment depth for H3K4me3 and H3K27ac marks in a ± 3 kb window.
4. Box plot showing the FPKM values for transcripts corresponding to ChIP-CTCF peaks common with NicE-seq and DNase-seq (bin II), common with NicE-seq peaks alone (bin III) and common with DNase-seq peaks alone (bin IV) from HCT116 RNAseq ENCODE data set.

**
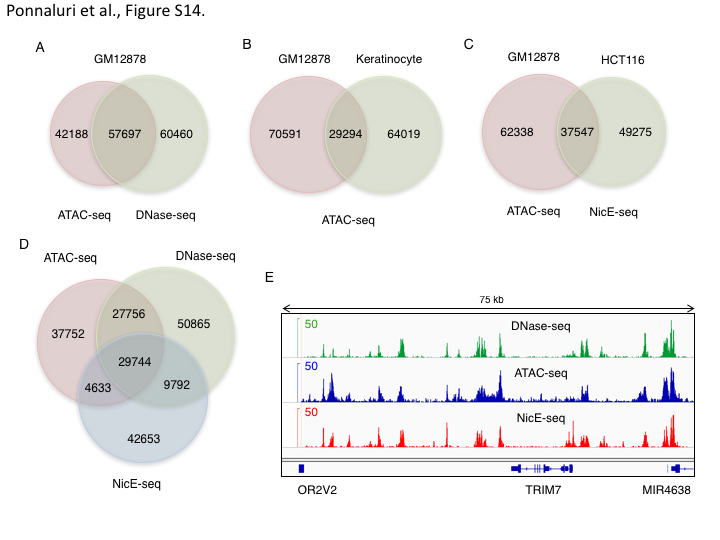
**

**Additional file1 Figure S14. Overlap of open chromatin peaks identified across different cell lines and with different methods.**

1. A Venn diagram showing the overlap between ATAC-seq and DNase-seq for GM12878 cells.
2. A Venn diagram showing the overlap between the open chromatin peaks identified by ATAC-seq for GM12878 and human primary neonatal keratinocytes.
3. A Venn diagram showing the overlap between the open chromatin peaks identified by ATAC-seq and NicE-seq for GM12878 and HCT116 respectively.
4. A Venn diagram showing the overlap between the open chromatin peaks identified by ATAC-seq, DNase-seq for GM12878 and NicE-seq for HCT116 cells.
5. Snapshot of IGV browser showing the overlap of OCS peaks (top panel in green for DNase-seq (HCT116), middle panel in blue for ATAC-seq (GM12878) and bottom panel in red for NicE-seq (HCT116)) in a window of 75 kb.

**
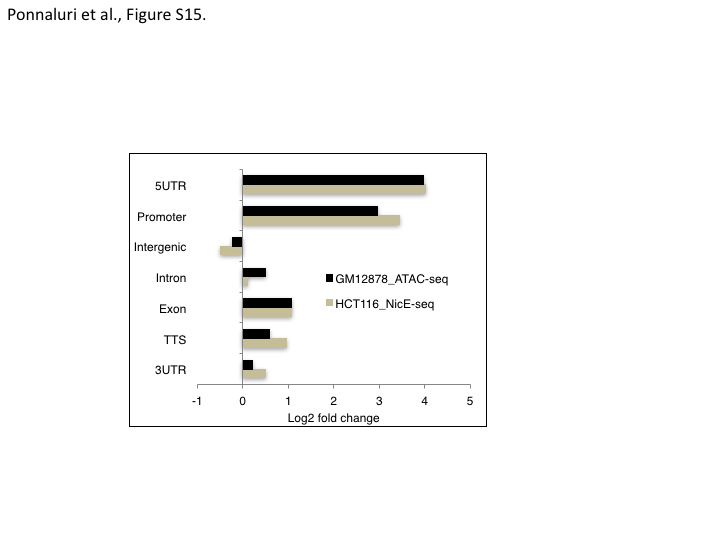
**

**Additional file1 Figure S15. Distribution of open chromatin peaks across different genomic regions.**

Bar graphs showing the log_2_ fold change of OCS peaks in different genomic regions identified by ATAC-seq for GM12878 and NicE-seq for HCT116 cells.

**
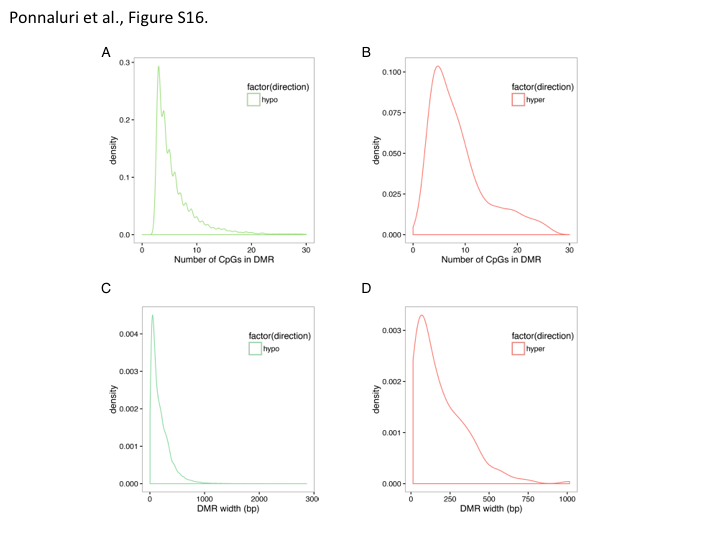
**

**Additional file1 Figure S16. Distribution of CpGs in the DMRs.**

1. Density plot showing the distribution of number of CpGs in the hypomethylated DMRs in green.
2. Density plot showing the distribution of number of CpGs in the hypermethylated DMRs in red.
3. Density plot showing the distribution of the hypomethylated DMRs width in green.
4. Density plot showing the distribution of the hypermethylated DMRs width in red.

**
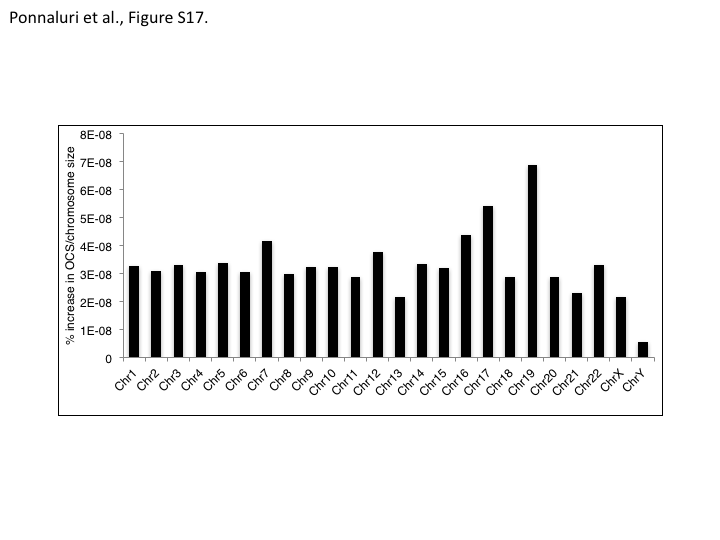
**

**Additional file1 Figure S17. Percentage increase in OCS peaks after 5-aza-2’deoxycytidine treatment.**

Bar graph showing the percentage increase in OCS peaks after 6 days of 5 µM 5-aza-2’-deoxycytidine treatment for all chromosomes normalized to the size of the chromosome.
